# Obesity Variants in the *GIPR* Gene Are not Associated With Risk of Fracture or Bone Mineral Density

**DOI:** 10.1210/clinem/dgad734

**Published:** 2023-12-20

**Authors:** Unnur Styrkarsdottir, Vinicius Tragante, Lilja Stefansdottir, Gudmar Thorleifsson, Asmundur Oddsson, Erik Sørensen, Christian Erikstrup, Peter Schwarz, Henrik Løvendahl Jørgensen, Jes Bruun Lauritzen, Søren Brunak, Kirk U Knowlton, Lincoln D Nadauld, Henrik Ullum, Ole Birger Vesterager Pedersen, Sisse Rye Ostrowski, Hilma Holm, Daniel F Gudbjartsson, Patrick Sulem, Kari Stefansson

**Affiliations:** Population Genomics, deCODE genetics/Amgen Inc, Reykjavik 102, Iceland; Population Genomics, deCODE genetics/Amgen Inc, Reykjavik 102, Iceland; Population Genomics, deCODE genetics/Amgen Inc, Reykjavik 102, Iceland; Population Genomics, deCODE genetics/Amgen Inc, Reykjavik 102, Iceland; Population Genomics, deCODE genetics/Amgen Inc, Reykjavik 102, Iceland; Department of Clinical Immunology, Copenhagen University Hospital, Rigshospitalet, Copenhagen 2100, Denmark; Department of Clinical Immunology, Aarhus University Hospital, Aarhus 8200, Denmark; Department of Clinical Medicine, Aarhus University, Aarhus 8200, Denmark; Department of Clinical Medicine, Faculty of Health and Medical Sciences, University of Copenhagen, Copenhagen 2200, Denmark; Department of Endocrinology, Copenhagen University Hospital, Rigshospitalet, Copenhagen 2100, Denmark; Department of Clinical Medicine, Faculty of Health and Medical Sciences, University of Copenhagen, Copenhagen 2200, Denmark; Department of Clinical Biochemistry, Amager Hvidovre Hospital, Copenhagen 2650, Denmark; Department of Orthopedic Surgery, Bispebjerg Hospital, University of Copenhagen, Copenhagen 2400, Denmark; Novo Nordisk Foundation Center for Protein Research, University of Copenhagen, Copenhagen 2200, Denmark; Intermountain Health, Heart Institute, Salt Lake City, UT 84143, USA; Intermountain Health, Cancer Center, St.George, UT 84790, USA; Statens Serum Institut, Copenhagen 2300, Denmark; Department of Clinical Medicine, Faculty of Health and Medical Sciences, University of Copenhagen, Copenhagen 2200, Denmark; Department of Clinical Immunology, Zealand University Hospital, Køge 4600, Denmark; Department of Clinical Immunology, Copenhagen University Hospital, Rigshospitalet, Copenhagen 2100, Denmark; Department of Clinical Medicine, Faculty of Health and Medical Sciences, University of Copenhagen, Copenhagen 2200, Denmark; Population Genomics, deCODE genetics/Amgen Inc, Reykjavik 102, Iceland; Population Genomics, deCODE genetics/Amgen Inc, Reykjavik 102, Iceland; School of Engineering and Natural Sciences, University of Iceland, Reykjavik 102, Iceland; Population Genomics, deCODE genetics/Amgen Inc, Reykjavik 102, Iceland; Population Genomics, deCODE genetics/Amgen Inc, Reykjavik 102, Iceland; Faculty of Medicine, School of Health Science, University of Iceland, Reykjavik 102, Iceland

**Keywords:** fractures, BMF, GIP, GIPR, association, burden

## Abstract

**Context:**

It is not clear if antagonizing the GIP (glucose-dependent insulinotropic polypeptide) receptor (GIPR) for treatment of obesity is likely to increase the risk of fractures, or to lower bone mineral density (BMD) beyond what is expected with rapid weight loss.

**Objective:**

The objective of this study was to investigate the risk of fracture and BMD of sequence variants in *GIPR* that reduce the activity of the GIP receptor and have been associated with reduced body mass index (BMI).

**Methods:**

We analyzed the association of 3 missense variants in *GIPR*, a common variant, rs1800437 (p.Glu354Gln), and 2 rare variants, rs139215588 (p.Arg190Gln) and rs143430880 (p.Glu288Gly), as well as a burden of predicted loss-of-function (LoF) variants with risk of fracture and with BMD in a large meta-analysis of up to 1.2 million participants. We analyzed associations with fractures at different skeletal sites in the general population: any fractures, hip fractures, vertebral fractures and forearm fractures, and specifically nonvertebral and osteoporotic fractures in postmenopausal women. We also evaluated associations with BMD at the lumbar spine, femoral neck, and total body measured with dual-energy x-ray absorptiometry (DXA), and with BMD estimated from heel ultrasound (eBMD).

**Results:**

None of the 3 missense variants in *GIPR* was significantly associated with increased risk of fractures or with lower BMD. Burden of LoF variants in *GIPR* was not associated with fractures or with BMD measured with clinically validated DXA, but was associated with eBMD.

**Conclusion:**

Missense variants in *GIPR*, or burden of LoF variants in the gene, are not associated with risk of fractures or with lower BMD.

The incretin hormonal system has recently been the focus of several treatment strategies for obesity and type 2 diabetes (1, 2). Incretins are glucose-dependent gut hormones that control metabolic physiology across many organs, including stimulation of insulin secretion after oral food intake, a phenomenon known as the incretin effect (3). Two incretins, GIP (glucose-dependent insulinotropic polypeptide) and GLP-1 (glucagon-like peptide-1), act through their binding to their cognate G protein–coupled receptors, GIPR and GLP-1R, which consequently stimulate downstream signaling processes. Both receptors have been assessed as potential therapeutic targets, either with agonists, antagonists, or in combination therapies (2).

Common sequence variants in the human *GIPR* gene, encoding GIPR, were found to be associated with lower body mass index (BMI) (4) as well as glycemic traits (5) in large meta-analyses, with follow-up experiments showing that the variants reduce the incretin effect (5), indicating reduced function of the GIPR protein. A representative of these common variants is a missense variant in the gene, rs1800437 (p.Glu354Gln). Two additional rare missense variants, rs139215588 (p.Arg190Gln) and rs143430880 (p.Glu288Gly), have also been found to be associated with lower BMI (6, 7) and to impair GIPR function (7). Furthermore, burden test of predicted rare loss-of-function (LoF) and predicted deleterious missense variants in *GIPR*, which result in nonfunctional receptor, are also associated with lower BMI (7). Since the receptor is less active in individuals who carry these sequence variants, their effect can therefore be considered to mimic GIPR antagonization. Hence, diminishing the activity of GIPR may be a way to treat obesity as individuals who carry these variants have considerably lower BMI than the general population.

Concerns have been raised that GIPR antagonism might increase the risk of fractures as one of the common missense variants, rs1800437 (p.Glu354Gln), has been reported to be associated with risk of fractures in postmenopausal women (8). rs1800437 increased the risk of incident nonvertebral fracture and was associated with lower hip bone mineral density (BMD) in a longitudinal study of 1424 postmenopausal Danish women (8). After 10 years of follow-up, carriers of the BMI-lowering rs1800437[C] allele (p.354Gln) were reported to have lower hip BMD (femoral neck [FN] and total hip) than noncarriers. After 16 years of follow-up, women homozygous for the C allele (rs1800437[CC]) had increased risk of nonvertebral fractures. None of the other 3 fracture categories that were evaluated (any fracture, osteoporotic fracture, and nonvertebral osteoporotic fracture) were associated with rs1800437, and neither was BMD in the lumbar spine (LS).

In this study we investigated the association of sequence variants in *GIPR* with fracture risk and BMD in a large, well-powered meta-analysis of fractures and BMD in 100 000 to 1.2 million participants. We not only tested rs1800437, but all 3 missense variants that have been shown to impair the function of GIPR (rs1800437(p.Glu354Gln), rs139215588 (p.Arg190Gln), and rs143430880 (p.Glu288Gly)) and to be associated with BMI, and rare variants that are predicted to be deleterious to the protein in a burden test. We also focus on postmenopausal women specifically for direct comparison with the previous Danish study (8).

## Material and Methods

### Study Populations

#### Iceland

The Icelandic fracture data set is derived from 3 sources: 1) a population data set of fractures (all types of fractures) obtained from Landspitali—the National University Hospital electronic health records from 1993 to 2017, and the Directorate of Health's from 2005 to 2018, which contains fracture information from all hospitals and primary care clinics in Iceland; 2) a registry of all hip fractures in Iceland from 1950 to 2010; and 3) questionnaires gathered at the deCODE genetics osteoporosis project and a general population screening project, the deCODE Health Study (9). There is considerable overlap between the 3 sets. The controls did not include individuals with fractures.

BMD was measured with densitometers at the Landspitali University Hospital's dual-energy x-ray absorptiometry (DXA) clinic and the Research Service Center's Heilsurannsokn DXA clinic, both using Hologic DXA machines. Values at the hip, the LS (L1-L4), and the total body (TB) were corrected for DXA machine used, and adjusted for age, height, and BMI and standardized for each sex separately to a mean = 0 and SD = 1.

Height, weight, and BMI information from participants was obtained by aggregating a) hospital records from Landspitali; b) general practitioners’ (GP) records; and c) multiple research studies conducted at deCODE genetics. In case of multiple different height measurements, the most common measurement (mode) was selected to represent each individual’s height. Multiple weight measurements were averaged. Association analyses were conducted on individuals older than 18 years using sex, year of birth, and age at first measurement as covariates.

All participants who donated biological samples gave informed consent, and the National Bioethics Committee of Iceland approved the study (VSN-15-198, VSN-15-214, VSN-17-076), which was conducted in agreement with conditions issued by the Data Protection Authority of Iceland.

#### United Kingdom

The UK Biobank resource (http://www.ukbiobank.ac.uk) includes data from 500 000 volunteer participants who were recruited between ages 40 and 69 years in 2006 to 2010 across the United Kingdom. All individuals in the present study were of White British or Irish descent (N = 431 805). For all participants, inpatient and outpatient health-care records and health-related information were collected and regularly updated. Fractures were identified by International Classification of Diseases, Tenth Revision (ICD10) codes from hospital inpatient records (HESIN) and from primary care records (GP). The controls did not include individuals with fractures.

DXA BMD measures were adjusted for age, height, and BMI for each sex separately and standardized to a mean = 0 and SD = 1.

BMI (field f.21001) was adjusted for year of birth, age, age squared, and 20 principal components (PCs) for male and female participants separately, then combined, average taken over multiple measurements for individuals, and then inverse normal-transformed.

All participants gave informed consent, and UK Biobank's scientific protocol and operational procedures were reviewed and approved by the North West Research Ethics Committee. This research has been conducted using the UK Biobank Resource under application numbers 23359 and 56270.

#### Denmark

The Danish data set is derived from the Copenhagen Hospital Biobank (CHB) osteoporosis study (10) and the Danish Blood Donor Study (DBDS) (11). CHB was initiated in 2009 and contains leftover EDTA whole-blood sample blood type and screen tests conducted at hospitals in the capital region of Denmark. DBDS is a large, prospective cohort of Danish blood donors. At inclusion, blood donors provide questionnaire data on health-related items, access to data from national health registers, and whole-blood samples from each donation. Blood donation and participation in DBDS is voluntary and unpaid. Fractures were identified by ICD10 codes from hospital records. Control individuals did not include any fracture cases.

BMI measurements were available only for the DBDS cohort. BMI was calculated based on self-reported height and weight in the DBDS questionnaire data, where all individuals were between ages 17 and 67 years when answering the survey. In the case of multiple height and/or weight measurements, the average BMI was taken for each individual. Association analyses were conducted using age, sex, and 20 PCs as covariates.

The present project is approved by the National Committee on Health Research Ethics and the Capital Region Data Protection Agency for CHB-PDS (NVK-1903714) and DBDS (NVK-1700407 and P-2019-99). Statistical analysis on Danish data was performed in parallel at deCODE genetics and at Computerome (www.computerome.dk).

#### United States

The samples from the United States were derived from the Intermountain Health HerediGene: Population Study and INSPIRE studies. Participants (male and female, aged ≥ 18 years, and a US resident) visiting an Intermountain Health facility (Utah, USA) or event were recruited for study participation. Individuals were informed of the study protocol and procedures prior to providing consent. Control participants did not include fractured cases. All individuals included in this study were genetically determined to be of European descent.

BMI measurements: In case of multiple height measurements, the most common measurement was selected to represent the individual's height. Multiple weight measurements were averaged. Association analyses were conducted on individuals older than 18 years using age, sex, year of birth, and 4 genetic PCs as covariates.

Study procedures were in accordance with the ethical standards of the responsible institution and approved by the institutional review board at Intermountain Health (HerediGene: IRB No. 1051071, and INSPIRE: IRB No. 1024811).

Characteristics of the study participants in each population are summarized in Supplementary Table S1 (12).

### Fracture Definitions

ICD10 codes, or equivalent ICD9 or ICD8 codes, for different types of fractures were as follows: All fractures S02, S12, S22, S32, S42, S52, S62, S72, S82, and S92, hip fracture S720, S721, vertebral fracture S220, S320, and distal-forearm/wrist fracture S620, S525, S526. Questionnaire data cover ankle, femur, hip, knee, lower arm, lower leg, rib, upper arm, and vertebral, wrist, or other fractures. Osteoporotic fractures were defined with ICD codes S220, S32, S422, S424, S720, S721, S722, S823, S825, and S826, and corresponding fractures from questionnaires, and nonvertebral fractures as S02, S222, S223, S224, S321, S322, S323, S324, S325, S42, S52, S62, S72, S82, S92, and corresponding fractures from questionnaires.

### Genotyping

The Icelandic, Danish, and US samples were genotyped by deCODE genetics, using Illumina HumanHap and HumanOmni chips for the Icelandic samples and Illumina GSA chip for the Danish and US samples. Samples with less than a 96% yield were excluded. For each sample set, variants were excluded if they had less than a 98% yield, failed the Hardy-Weinberg test (*P* < 1 × 10^−6^), or showed a statistically significant (*P* < 1 × 10^−6^) difference between genotype batches. The UK Biobank genotyping was performed using a custom-made Affimetrix chip, UK BiLEVE Axiom (13), and with the Affimetrix UK Biobank Axiom array (14).

In the Icelandic samples, variants were derived from whole-genome sequencing (WGS) of 63 460 Icelanders using GAIIx, HiSeq, HiSeqX, and NovaSeq Illumina technology (15, 16), the genotypes of single-nucleotide variations (SNVs) and indels called jointly by GraphTyper (17), haplotyped long-range phased (18) and high-quality sequence variants imputed into all samples.

The variants imputed into the UK Biobank samples were derived from WGS of 131 958 UK individuals, performed jointly by deCODE genetics and the Welcome Trust Sanger Institute (19), where more than 245 million high-quality sequence variants and indels were identified using GraphTyper (17). Quality-controlled chip genotype data were phased using SHAPEIT 4 (20). A phased haplotype reference panel was prepared from the sequence variants using the long-range phased, chip-genotyped samples using inhouse tools and methods described previously (15, 16) and imputed into the phased genotype data.

The variants in the US and Danish samples were derived from WGS sequencing 10 828 individuals of non-Icelandic northern European descent, 464 million variants in total, long-range phased using SHAPEIT4 (20) and imputed into the chip data.

### Ancestry Analysis

For UK Biobank we used only data from the British-Irish ancestry group (N = 431 805) as previously defined (19). For this group 20 PCs were calculated and included in the association analysis to adjust for remaining population structure. Danish and US samples were excluded if they were identified as ethnic outliers in the respective cohort by ADMIXTURE (v 1.2) (21) and EIGENSOFT (v 6.0.1) (22) software, and to adjust for remaining population substructure PCs were included as covariates in the subsequent association analysis.

### Association Analysis

Logistic regression was used to test for association between variants and disease, assuming an additive/multiplicative model, treating disease status as the response and expected genotype counts from imputation as covariates. Testing was performed using the likelihood ratio statistic using software developed at deCODE genetics (15). For Iceland we included county of birth, age, age squared, sex, and an indicator function for the overlap of the lifetime of the individual with the time span of phenotype collection as covariates to account for differences between cases and controls. We used county of birth as a proxy covariate for the first PCs in our analysis because county of birth has been shown to be in concordance with the first PC in Iceland (23). The UK, Danish, and US associations were adjusted for sex, age, and the first 20 PCs.

We used linkage disequilibrium score regression (24) to account for distribution inflation due to cryptic relatedness and population stratification in all of the cohorts.

### Fracture Meta-Analyses

We meta-analyzed genome wide association study summary results from the Icelandic, Danish, US, and the UK samples under both the additive and recessive models using a fixed-effects inverse variance method (25).

### Bone Mineral Density Meta-Analyses

We downloaded summary statistics from a meta-analysis of LS BMD and FN BMD from the Genetic Factors for Osteoporosis Consortium (GEFOS) that did not include Icelandic data (26), and meta-analyzed these with the summary statistics from Iceland and UK Biobank. We also downloaded summary statistics of a meta-analysis of TB BMD from the GEFOS consortium (27) that includes a subset of the Icelandic data. For analysis of rs1800437, we meta-analyzed the TB GEFOS data with summary statistics from UK Biobank only as the Icelandic data set was included in the GEFOS data, but with both UK Biobank and Icelandic data for the rare variants as these are not present in the GEFOS TB data.

For eBMD (estimated BMD from heel ultrasound), we looked up the variants in eBMD results from UK Biobank. The eBMD was calculated as described in Morris et al (28) and adjusted for covariates for each sex separately.

### Burden Test

We used Variant Effect Predictor (VEP) (29) to predict consequences to the variants sequenced in each data set. We classified as high-impact variants those predicted as start-lost, stop-gain, stop-lost, splice donor, splice acceptor or frameshift, collectively called loss-of-function (LoF) variants. We classified as moderate-impact variants those missense variants predicted with LoF by 2 CADD (Combined Annotation Dependent Depletion) with a score greater than or equal to 25 (30), using variants available in dbNSFP v4.1c (31) (collectively called MIS). To allow for a comparable model to Akbari et al (7), we added variant rs139215588 despite its lower CADD score (22.1).

For case-control analyses, we used logistic regression under an additive model to test for association between 1) LoF or 2) LoF + MIS gene burdens and phenotypes, in which disease status was the dependent variable and genotype counts as the independent variable, using the likelihood ratio test to compute 2-sided *P* values. Individuals were coded 1 if they carry any of the LoF variants (LoF/LoF + MIS) with minor allele frequency less than 2% in the autosomal gene being tested and 0 otherwise. The same covariates used in the single-variant analyses were also used for gene burden analyses. Quantitative traits were inverse-normal–transformed and analyzed using a linear mixed-model implemented in BOLT-LMM (32). For the analyses, we used software developed at deCODE genetics (15). We used linkage disequilibrium score regression intercepts (24) to adjust the χ^2^ statistics and avoid inflation due cryptic relatedness and stratification, using a set of 1.1 million variants. *P* values were calculated from the adjusted χ^2^ results.

Meta-analysis was performed on the summary results from Iceland, the United Kingdom, Denmark, and the United States, when available, using a fixed-effects inverse variance-weighted method (25), in which the data sets were allowed to have different population frequencies for alleles and genotypes but were assumed to have a common odds ratio (OR) and weighted with the inverse of the variance of the effect estimate derived from the logistic regression.

### Selection of Individual Sequence Variants

We selected the individual SNVs rs1800437 (p.Glu354Gln), rs139215588 (p.Arg190Gln), and rs143430880 (p.Glu288Gly) for assessment based on previous results on their associations with BMI (4, 6) and because of the association of rs1800437 with incident fracture risk in postmenopausal Danish women (8).

## Results

### rs1800437 (p.Glu354Gln), rs139215588 (p.Arg190Gln), and rs143430880 (p.Glu288Gly) Were Associated With Body Mass Index

To quality-control the data sets that we used for evaluation of fracture risk (Iceland, Denmark [CHB-DBDS], United Kingdom [UK Biobank], and the United States [Intermountain Health]), we first assessed the association of the 3 missense variants in *GIPR* that have been shown to be associated with BMI in 629 233 individuals from these sample sets.

All 3 variants were associated with BMI with effects consistent with previous reports (4, 6, 7) (Table 1 and Supplementary Table S2 (12)).

**Table 1.** Association of missense variants in the *GIPR* gene with body mass index

Variant	Pos_build38	EA/NEA	Freq. EA %	Model	*P*	Effect (95% CI)	*P*_het_
rs1800437	19:45678134	C/G	20.9	A	4.8E-43	−0.033 (−0.038 to −0.028)	.07
				R	5.6E-19	−0.057 (−0.070 to −0.044)	.23
rs139215588	19:45674762	A/G	0.12	A	7.7E-06	−0.117 (−0.168 to −0.066)	.15
				R	NA	NA	NA
rs143430880	19:45677718	G/A	0.12	A	6.3E-07	−0.123 (−0.171 to −0.075)	.09
				R	NA	NA	NA

The 3 SNVs, rs1800437[C/G] (p.Glu354Gln), rs139215588[A/G] (p.Arg190Gln), and rs143430880[G/A] (p.Glu288Gly), were evaluated for association with body mass index in 629 233 individuals from the 4 populations used in the fracture study: Iceland, United States, and Denmark. The association model, *P* values, and effect in SD with 95% CIs are shown. *P*_het_ is the heterogeneity *P* value. A is additive model and R is recessive model.

Abbreviations: NA, not available; SNV, single-nucleotide variation.

### rs1800437 (p.Glu354Gln), rs139215588 (p.Arg190Gln), and rs143430880 (p.Glu288Gly), Were not Associated With Increased Risk of Fractures

We assessed the association of the 3 missense variants in *GIPR* with fractures in a case-control meta-analysis of postmenopausal women from Iceland, Denmark (CHB-DBDS), United Kingdom (UK Biobank), and the United States (Intermountain Health), including up to 105 000 fractured postmenopausal women and 350 000 nonfractured postmenopausal controls (see Supplementary Table S1 (12)). We evaluated association with nonvertebral fractures, vertebral fractures, and osteoporotic fractures as defined in Torekov et al (8), but also included hip fractures. We also assessed association with fractures in the general population, not restricting to postmenopausal women. The major fracture groups included in the analysis were hip fractures, vertebral fractures, forearm/wrist fractures, as well as any fractures.

We considered both an additive/multiplicative and recessive model of risk for association of rs1800437. Only the additive/multiplicative model could be tested for the 2 rare variants since these are too low in frequency to observe enough homozygotes of the rare alle.

In the postmenopausal group, the BMI-lowering rs1800437[C] allele (4, 33) was associated nominally with reduced risk of nonvertebral (*P* = 1.1 × 10^−3^; OR = 0.98), osteoporotic (*P* = 1.8 × 10^−4^; OR = 0.97), and hip fractures (*P* = .03; OR = 0.95) under the additive model, and rs143430880[G] was associated nominally with risk of osteoporotic fractures (*P* = .02; OR = 1.32) (Table 2 and Supplementary Table S3 (12)). rs1800437 was not associated with any fracture type under the recessive model in postmenopausal women.

**Table 2.** Association of the 3 body mass index missense variants in the *GIPR* gene with fractures

				rs1800437[C]—21.4%		rs139215588[A]—0.12%		rs143430880[G] −0.13%	
Group	Fracture	N cases/N controls	Model	*P*	OR (95% CI)	*P*	OR (95% CI)	*P*	OR (95% CI)
**Postmenopausal**	**Nonvertebral**	105 739/350 006	A	.001	0.98 (0.96-0.98)	.41	0.99 (0.98-1.01)	.15	1.12 (0.96-1.30)
			R	.12	0.97 (0.93-1.01)	NA	NA	NA	NA
	**Osteoporotic**	52 277/350 005	A	1.8E-4	0.97 (0.95-0.98)	.60	1.11 (0.75-1.63)	.02	1.32 (1.05-1.65)
			R	.12	0.96 (0.91-1.01)	NA	NA	NA	NA
	**Hip**	21 046/340 503	A	.03	0.95 (0.92-1.00)	.57	1.14 (0.73-1.80)	.37	1.27 (0.75-2.14)
			R	.40	0.97 (0.90-1.04)	NA	NA	NA	NA
	**Vertebral**	7942/340 548	A	.21	0.98 (0.96-1.01)	.15	0.89 (0.75-1.04)	.06	1.42 (0.99-2.01)
			R	.34	0.95 (0.84-1.06)	NA	NA	NA	NA
**All**	**Any type**	352 343/906 092	A	.11	0.99 (0.99-1.00)	.24	1.06 (0.96-1.18)	.20	1.06 (0.97-1.16)
			R	.08	0.98 (0.96-1.00)	NA	NA	NA	NA
	**Hip**	34 127/896 691	A	.20	0.99 (0.97-1.01)	.97	0.99 (0.72-1.38)	.09	1.24 (0.97-1.60)
			R	.43	0.98 (0.92-1.04)	NA	NA	NA	NA
	**Forearm/wrist**	79 986/851 397	A	.61	1.00 (0.98-1.01)	.41	1.08 (0.90-1.31)	.20	1.11 (0.95-1.30)
			R	.07	0.97 (0.93-1.00)	NA	NA	NA	NA
	**Vertebral**	18 473/869 228	A	.05	0.97 (0.95-1.00)	.03	1.44 (1.04-1.99)	.65	1.08 (0.76-1.54)
			R	.41	0.97 (0.90-1.04)	NA	NA	NA	NA

The 3 SNVs, rs1800437[C/G] (p.Glu354Gln), rs139215588[A/G] (p.Arg190Gln), and rs143430880[G/A] (p.Glu288Gly), were evaluated for association with fractures in postmenopausal women, and in all participants, irrespective of sex and age. Shown are the type of fracture, number (N) of cases and controls, the association model, *P* values, and ORs with 95% CIs. A is additive model and R is recessive model.

Abbreviations: NA, not available; OR, odds ratio; SNV, single-nucleotide variation.

In the general population, rs1800437[C] was not associated with fractures under the additive nor the recessive model. The rare variant rs139215588[A] was associated nominally with risk of vertebral fracture (*P* = .03; OR = 1.44).

Among the previously described variants, only the association of rs1800437 with reduced risk of nonvertebral fractures and osteoporotic fractures in postmenopausal women survives correction for multiple testing (*P*_Bonferroni_ < .002) but the effect is in the opposite direction of that reported in the previous Danish study.

### rs1800437 (p.Glu354Gln), rs139215588 (p.Arg190Gln) and rs143430880 (p.Glu288Gly), Were Not Associated With Bone Mineral Density

We assessed associations of the 3 missense variants in *GIPR* with BMD of LS, FN, and TB in our large meta-analysis of Icelandic, UK, and GEFOS consortium (26) data, and with eBMD in the UK Biobank.

rs1800437[C] was associated with increased LS BMD (*P* = 1.4 × 10^−4^; effect = 0.022 SD; Bonferroni threshold for significance = 0.004), but not with FN, TB, or eBMD (Table 3 and Supplementary Table S3 (12)). It was not associated with any BMD site under a recessive model. The 2 rare variants were not associated with any of the BMD phenotypes tested.

**Table 3.** Association of the 3 body mass index missense variants in the *GIPR* gene with bone mineral density

BMD	N individuals	Model	rs1800437[C]—21.4%		rs139215588[A]—0.12%		rs143430880[G] −0.13%	
			*P*	Effect (95% CI)	*P*	Effect (95% CI)	*P*	Effect (95% CI)
Lumbar spine	102 184	A	.0001.4E-4	0.022 (0.011 to 0.033)	.38	−0.056 (−0.179 to 0.068)	.27	−0.092 (−0.253 to 0.070)
	74 539	R	.07	0.035 (−0.003 to 0.072)	NA	NA	NA	NA
Femoral neck	101 082	A	.39	0.005 (−0.006 to 0.015)	.66	−0.027 (−0.148 to 0.094)	.41	−0.067 (−0.227 to 0.093)
	75 650	R	.44	−0.015 (−0.052 to 0.023)	NA	NA	NA	NA
Total body	102 021	A	.04	0.012 (0.000 to 0.023)	.35	0.065 (−0.072 to 0.202)	.23	−0.097 (−0.256 to 0.061)
	55 693	R	.84	0.004 (−0.038 to 0.046)	NA	NA	NA	NA
eBMD	398 823	A	.19	−0.004 (−0.010 to 0.002)	.20	−0.041 (−0.104 to 0.022)	.02	−0.066 (−0.121 to −0.011)
	398 823	R	.04	−0.016 (−0.031 to −0.001)	NA	NA	NA	NA

The 3 SNVs, rs1800437[C/G] (p.Glu354Gln), rs139215588[A/G] (p.Arg190Gln), and rs143430880[G/A] (p.Glu288Gly), were evaluated for association with BMD at different skeletal sites, with number of individuals (N) shown, the association model, *P* value, effect in SDs, and 95% CI.

Abbreviations: BMD, bone mineral density; eBMD, BMD estimated from heel ultrasound; NA, not available; SNV, single-nucleotide variation.

### Individuals Homozygous for the *GIPR* Rare Missense Variants, rs139215588 (p.Arg190Gln) and rs143430880 (p.Glu288Gly)

The recessive model could not be assessed for the 2 rare missense variants in *GIPR* because of their low frequency. Nobody was homozygous for rs13921558[A] in the entire data set. However, 3 individuals were homozygous for rs143430880[G], 2 in the UK Biobank and 1 in the Danish data set (2 women and and 1 man, between ages 62 and 66 years). Only one has suffered a fracture, an ankle fracture in a high-trauma accident at age 48 years. We did have BMI information on the 2 individuals from the United Kingdom but not the Danish one. Of note is their low BMI, −0.98 SD and −1.4 SD below the mean of the UK population.

### Burden Test of Rare Loss-of-Function and Deleterious Missense Variants in *GIPR*

We assessed results from burden tests of BMI in same sample sets as included in the fracture analysis. We assessed the burden of LoF variants, and LoF variants combined with missense variants that are predicted to be deleterious to the function of the GIPR protein (LoF + MIS).

LoF + MIS were significantly associated with BMI (effect = −0.11 SD; *P* = 4.6 × 10^−14^; Table 4 and Supplementary Table S5 (12)), whereas the LoF model was not (effect = −0.04 SD; *P* = .39; see Supplementary Table S5 (12)). The LoF variants in *GIPR* are very rare (between 0.0017% and 0.023% [Supplementary Table S6]) (12), and therefore our power to detect an association with them is limited.

**Table 4.** Burden of loss-of-function and missense variants

		OR (95% CI)	*P*	*P*_het_
**Postmenopausal**	Nonvertebral fracture	1.05 (0.83 to 1.32)	.68	.58
	Osteoporotic fracture	1.19 (1.05 to 1.35)	.008	.38
	Vertebral fracture	0.69 (0.34 to 1.39)	.30	.77
	Hip fracture	0.91 (0.55 to 1.50)	.71	.71
**All**	Any type of fracture	1.03 (0.99 to 1.08)	.19	.40
	Forearm/wrist fracture	1.04 (0.95 to 1.14)	.40	.31
	Hip fracture	1.30 (1.03 to 1.64)	.03	.92
	Vertebral fracture	1.16 (0.98 to 1.38)	.09	.37
		**Effect (95% CI)**	***P***	***P*_het_**
**BMI and BMD**	BMI	−0.110 (−0.139 to −0.081)	4.6E-14	.19
	Femoral neck BMD	0.020 (−0.055 to 0.095)	.60	≥.999
	Lumbar spine BMD	0.000 (0.000 to 0.000)	.95	.89
	Total body BMD	0.010 (−0.094 to 0.114)	.85	.87
	eBMD	−0.061 (−0.098 to −0.024)	.001	na

Results from burden test that includes predicted loss-of-function variants in *GIPR* and missense variants that are predicted to be deleterious to the protein. Results from the individual sample sets and for predicted loss of function variants only are shown in Supplementary Table S5 (12), and burden test characteristics per sample set are shown in Supplementary Table S6 (12). Shown are fracture types in postmenopausal women, and in all individuals irrespective of age and sex, along with ORs and 95% CIs. For BMI and BMD phenotypes, the effect is shown in SDs. *P*_het_ is the heterogeneity *P* value.

Abbreviations: BMD, bone mineral density; BMI, body mass index; eBMD, BMD estimated from heel ultrasound; OR, odds ratio; SNV, single-nucleotide variation.

We then tested for an association between LoF and LoF + MIS burden with fractures and BMD. Burden of LoF variants was not associated with any of the fracture phenotypes nor BMD at any site. LoF + MIS burden was nominally associated with greater risk of hip fractures and postmenopausal osteoporotic fractures (*P* = .03 and *P* = .008, respectively), as was the association with lower eBMD (*P* = .001). However, only the association with lowered eBMD remained statistically significant after accounting for multiple testing (*P* < .002).

## Discussion

Here we show that the alleles of the 3 missense variants in *GIPR* that are associated with reduced BMI are not associated with increased risk of fractures or lower BMD in a large meta-analysis of 100 000 to 1.2 million individuals. Neither were burden tests of predicted LoF variants or missense variants in the *GIPR* gene that are predicted to be deleterious to the protein.

Previously, Torekov et al (8) found that the common (21% minor allele frequency) missense variant in the *GIPR* gene, rs1800437 (p.Glu354Gln), which is associated with lower BMI, was also associated with lower hip BMD after 10 years of follow-up of women who were perimenopausal at the beginning of the study, and with risk of incident nonvertebral fractures after 16 years of follow-up. The fracture risk was significant only in women homozygous for the C risk allele compared with women homozygous for the major G allele. No association was observed with the other fracture groups tested (vertebral, osteoporotic, or any type), or with LS BMD, in this relatively small sample set of 1424 Danish postmenopausal women at the study end point, of whom 473 had suffered any type of fracture in the study period (first fracture).

We analyzed the association of rs1800437 with nonvertebral fractures in a much larger sample set of 105 739 postmenopausal women with a fracture compared to 350 006 nonfractured female controls, of whom approximately 25% are Danish. In this case-control setting we observed a nominal association of rs1800437[C] with reduced, and not increased, risk of nonvertebral fractures under the additive model, but not under the recessive model. Likewise, we observed a reduced risk of osteoporotic fractures in 52 277 patient cases and 350 005 controls under the additive model, but not under the recessive model. The prior study followed women who recently entered perimenopause to a fracture study end point 16 years later, ending at a mean age of approximately 66 years. In our case-control study of postmenopausal women, the mean age was between 65 years and 68 years, very comparable to the age at fracture end point in the prior study, enabling us to directly compare the results between the two studies despite differences in study setup. Both our study and the Danish study only included individuals of European descent.

Our further investigations involving a larger data set including all fractured individuals, irrespective of sex or age, also showed that rs1800437[C] does not confer risk of fracture. Furthermore, we did not replicate the association of rs1800437[C] with lower hip BMD in our large meta-analysis of 101 000 individuals, but rather showed an association with a modest increase of LS BMD.

We did not find an association of the other two rare missense variants in the *GIPR* gene with risk of fractures or with BMD. These variants are too rare to evaluate an association under a recessive model. We found only 3 homozygous carriers of rs143430880 but not 1 homozygous for rs139215588. Only 1 of the 3 individuals had suffered a fracture, and it was caused by high-energy trauma, indicating that a presumably near-complete LoF of the gene does not confer dramatic increase in fracture risk. Of note is, however, the low BMI of the 2 homozygous individuals on whom we had information on BMI measures, which is consistent with an earlier observation of the association between this variant and BMI.

Burden test of predicted LoF and deleterious missense variants (LoF + MIS) was associated with lower BMI, but not with fractures. It was associated also with lower eBMD (estimated BMD from heel ultrasound), but not with clinically validated BMD measurements from DXA scans.

Based on our observations, we conclude that sequence variants that reduce the activity of the GIPR protein are not associated with fractures or BMD in very large data sets of European descent.

## Data Availability

The data supporting the findings of this study are available within the article, its supplementary data files, and on request.

## Abbreviations

BMD: bone mineral density
BMI: body mass index
CADD: Combined Annotation Dependent Depletion
CHB: Copenhagen Hospital Biobank
DBDS: Danish Blood Donor Study
DXA: dual-energy x-ray absorptiometry
eBMD: BMD estimated from heel ultrasound
FN: femoral neck
GEFOS: Genetic Factors for Osteoporosis Consortium
GIP: glucose-dependent insulinotropic polypeptide
GLP-1: glucagon-like peptide-1
ICD10: International Classification of Diseases, Tenth Revision
LoF: loss-of-function
LS: lumbar spine
MIS: missense variants available in dbNSFP v4.1c that are predicted to lead to loss of function
OR: odds ratio
PC: principal component
SNV: single-nucleotide variation
TB: total body
WGS: whole-genome sequencing
